# Association of zoonotic protozoan parasites with microplastics in seawater and implications for human and wildlife health

**DOI:** 10.1038/s41598-022-10485-5

**Published:** 2022-04-26

**Authors:** Emma Zhang, Minji Kim, Lezlie Rueda, Chelsea Rochman, Elizabeth VanWormer, James Moore, Karen Shapiro

**Affiliations:** 1 Department of Pathology, Microbiology, and Immunology, School of Veterinary Medicine, University of California, Davis, CA USA; 2 Department of Ecology and Evolutionary Biology, University of Toronto, Toronto, ON Canada; 3 School of Veterinary Medicine and Biomedical Sciences, School of Natural Resources, University of Nebraska, Lincoln, NE USA; 4 Bodega Marine Laboratory, University of California, Davis, CA USA

**Keywords:** Microbial ecology, Environmental impact, Pathogens

## Abstract

Plastics are widely recognized as a pervasive marine pollutant. Microplastics have been garnering increasing attention due to reports documenting their ingestion by animals, including those intended for human consumption. Their accumulation in the marine food chain may also pose a threat to wildlife that consume species that can accumulate microplastic particles. Microplastic contamination in marine ecosystems has thus raised concerns for both human and wildlife health. Our study addresses an unexplored area of research targeting the interaction between plastic and pathogen pollution of coastal waters. We investigated the association of the zoonotic protozoan parasites *Toxoplasma gondii*, *Cryptosporidium parvum*, and *Giardia enterica* with polyethylene microbeads and polyester microfibers. These pathogens were chosen because they have been recognized by the World Health Organization as underestimated causes of illness from shellfish consumption, and due to their persistence in the marine environment. We show that pathogens are capable of associating with microplastics in contaminated seawater, with more parasites adhering to microfiber surfaces as compared with microbeads. Given the global presence of microplastics in fish and shellfish, this study demonstrates a novel pathway by which anthropogenic pollutants may be mediating pathogen transmission in the marine environment, with important ramifications for wildlife and human health.

## Introduction

Each year, millions of tons of plastic waste contaminate the world’s oceans^1^. Over time, plastic debris breaks down to microplastics, defined as plastics < 5 mm in size^2^. Microplastics have been garnering increasing attention due to reports documenting their ingestion by fish and marine invertebrates such as shellfish, including those intended for human consumption^3–5^. We are beginning to gain a better understanding about the potential consequences of microplastics to wildlife health^6,7^. We know much less about the consequences of microplastic consumption from seafood on human health. Potential health impacts may occur due to chemicals associated with the particles, pathogens that adhere to their surfaces, or physical impaction caused by the particles^8^.

Microplastic accumulation in the marine food chain may pose a threat to marine wildlife that consume lower trophic species that can accumulate these particles. Microplastics have been documented in multiple marine megafauna species such as sea turtles, beluga whales, and northern fur seals^9–11^. Plastic pollutants have even been found in Gentoo penguins of the South Orkney Islands, indicating that these particles have contaminated waters as remote as the Antarctic^12^. Although there have been increasing reports of mortalities in many marine wildlife species due to entanglement and gastric impaction from large plastic debris, we are just beginning to gain greater insight into the direct or indirect consequences of microplastic ingestion on lower trophic species that form the foundation of marine foodwebs^6,13^. One study observed an increase in the incidence of physical deformities in larval fathead minnows exposed to microplastics collected from the shorelines of Lake Ontario^14^. There is also empirical evidence demonstrating trophic transfer as an indirect route of microplastic ingestion for top marine predators^15^. Microplastic contamination in marine ecosystems has thus raised concerns for both wildlife and human health^16^.

Although it is clear that microplastics can lead to adverse health effects, more research is needed to understand the mechanism(s) that lead to these effects^6^. One notable question is whether microplastics can mediate transport of aquatic contaminants that can adhere to their surfaces. While some studies have reported on the ability of plastic debris to bind to and transport chemical pollutants^17^, little research has investigated the ability of microplastics to mediate the transport and fate of biological contaminants such as pathogens. Prior studies have demonstrated that unique communities of bacteria colonize plastic debris surfaces compared to the bacterial communities in the surrounding seawater, and these communities have been since dubbed the “Plastisphere”^18^. These plastic associated biofilms have also been found to carry unique fungal communities as well as harmful algal species^19^. Few studies have described an association between microplastics and pathogenic microorganisms and have primarily focused on halophilic bacteria such as *Vibrio* spp^18,20^. To date, there have been no studies that report the interactions between microplastics and terrestrially derived pathogens including zoonotic protozoan parasites. While halophilic bacteria are capable of multiplying in the marine environment, the transmission of terrestrial protozoa is entirely dependent on factors that affect their transport, fate, and survival. The potential ability of microplastics to physically associate with these terrestrial pathogens would shed important insight on their transmission through the marine environment, with relevance to both wildlife and human health.

The purpose of this study was to investigate the association of the zoonotic protozoa *Toxoplasma gondii, Cryptosporidium parvum,* and *Giardia enterica* with microplastic surfaces. We selected these pathogens because they have been recognized by the World Health Organization as underestimated causes of illness from shellfish consumption in humans^21^. These protozoa have also been found to be persistent in seawater^22,23^ and have been reported as prevalent contaminants of commercial shellfish worldwide^24–26^. These pathogens can also cause illness in aquatic wildlife, and *T. gondii* infections in particular are widely prevalent in marine mammal populations worldwide^27^. Due to the ubiquitous nature of microplastics and protozoan pathogen pollution in seawater, the goal of this study was to evaluate the potential interaction between these pollutants. We conducted bench scale experiments to test the hypothesis that zoonotic protozoa can associate with microplastics in contaminated seawater. We further quantified and compared protozoan parasite association with two primary types of microplastics: polyethylene microbeads and polyester microfibers.

## Results

### Experiment 1: association of protozoan pathogens with microplastics over time

**Figure 1:**
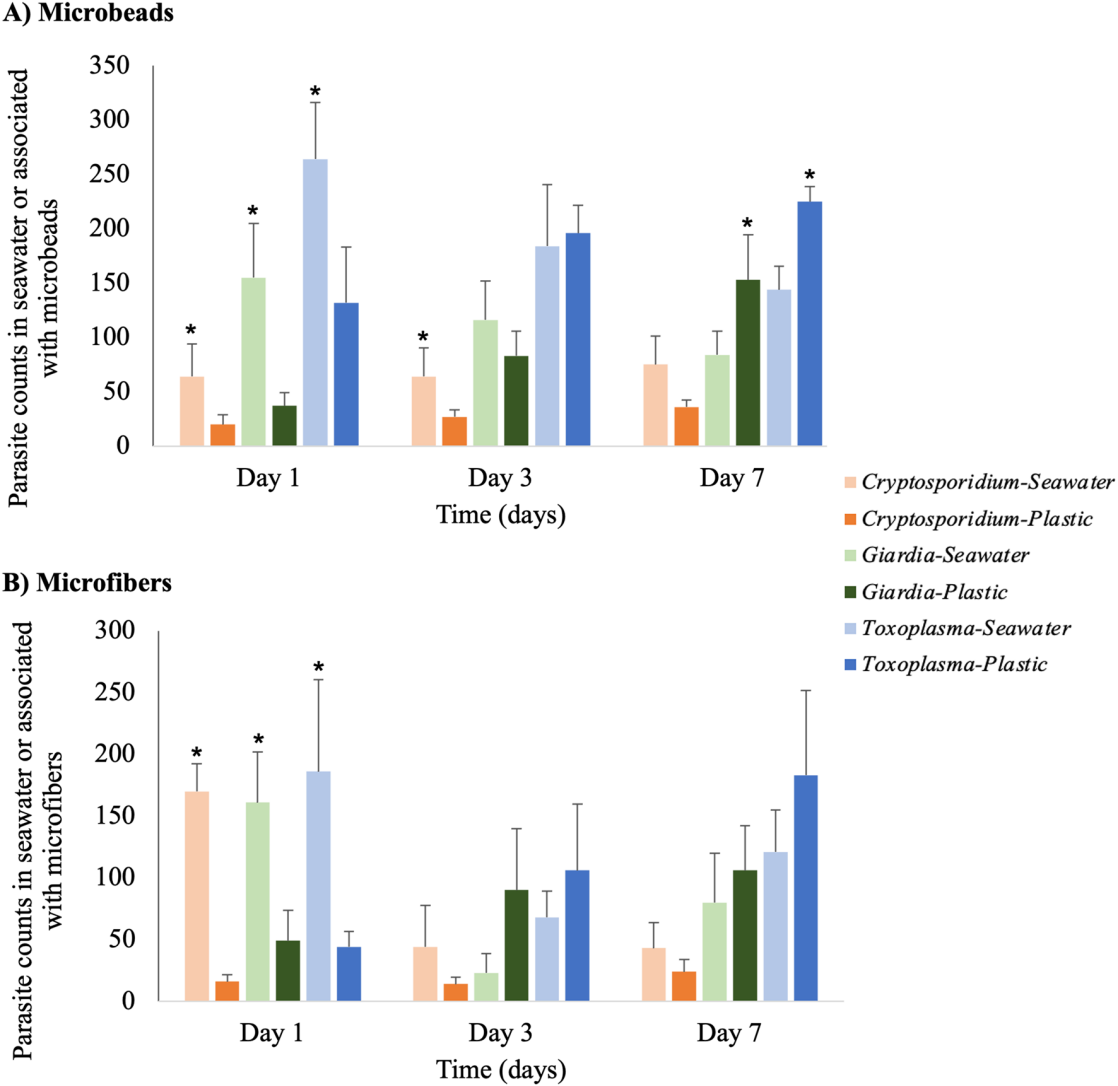
Experiment 1: Parasite (oo)cyst counts in surrounding seawater and associated with 500 μm polyethylene microbeads (**A**) or 800–1200 μm polyester microfibers (**B**). Lighter colors represent parasite counts in the seawater fraction while darker colors represent plastic associated counts. Error bars indicate one standard deviation from the mean. Asterisks indicate significance (*P* < 0.05) between parasite counts in seawater and microplastics.

All three selected protozoan parasites were associated with the surfaces of microplastics for both the microbeads and the microfibers (Fig. 1). For microbeads, the counts of parasites on these plastics increased over time. Conversely, parasite counts in seawater decreased over a 7-day period for *G. enterica* and *T. gondii*, but not for *C. parvum* (Fig. 1A). Counts of *G. enterica* that were associated with the microbeads increased significantly (*P* < 0.05) over each day of testing. *T. gondii* counts were significantly higher on the microbeads on day 7 compared with day 1, while *T. gondii* counts in the seawater were significantly lower on day 7 than day 1.

A similar trend was observed with the microfibers, with parasite counts associated with the microfibers generally increasing over time, with the exception of *C. parvum* from day 1 to day 3. Likewise, parasites in surrounding seawater decreased in counts between day 1 and 7 (Fig. 1B). Interestingly, in the microfiber experiment, we also observed an overall lower recovery of parasites in all samples collected on day 3 (Table S2). *G. enterica* counts in seawater decreased significantly over time. *C. parvum* counts in seawater decreased significantly between day 1 and 3 as well as day 1 and 7. The change over time in counts of *G. enterica* and *C. parvum* associated with microfibers was not statistically significant. The counts of *T. gondii* in seawater decreased significantly between day 1 and 3 while those associated with the microfibers increased significantly between day 1 and 7.

### Experiment 2: effect of microplastic type and size on association with protozoa

**Figure 2:**
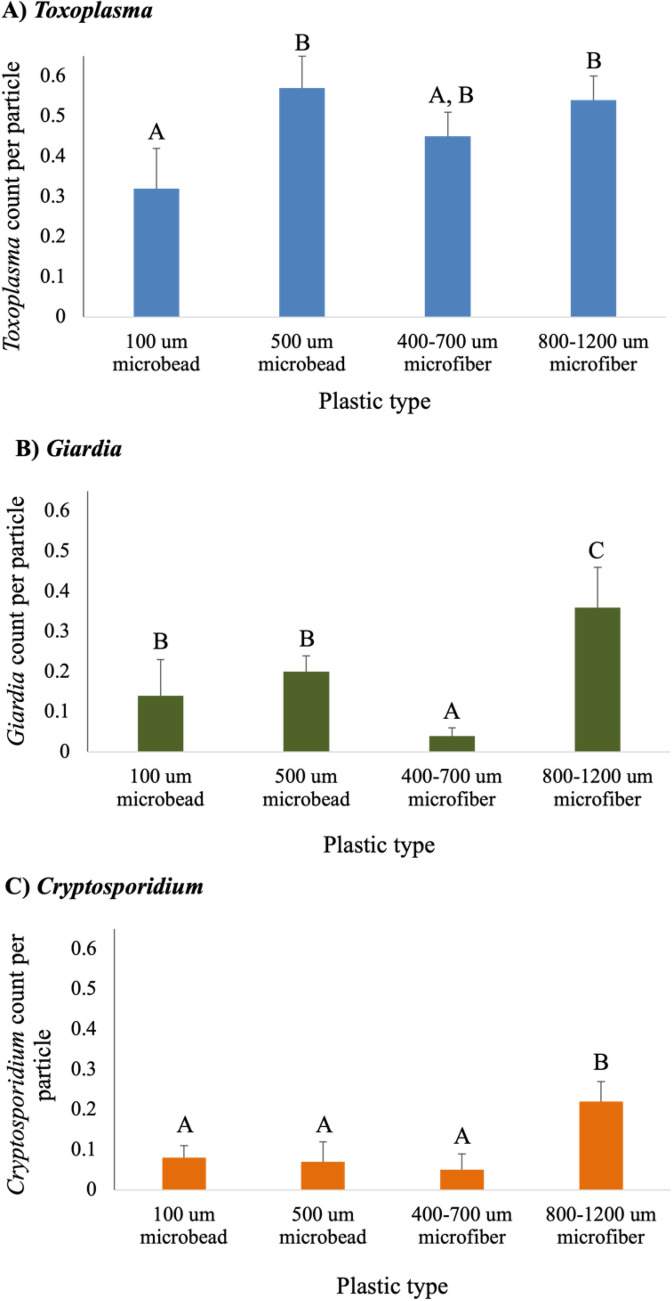
Experiment 2: Parasite (oo)cyst counts associated with four different microplastics (100 μm and 500 μm microbeads, as well as 400–700 μm and 800–1200 μm microfibers) in spiked seawater. Letters over each column indicate significant differences among parasite counts on microplastics across the different particle types. Samples that do not share a letter in common are statistically different (*P* < 0.05).

**Figure 3:**
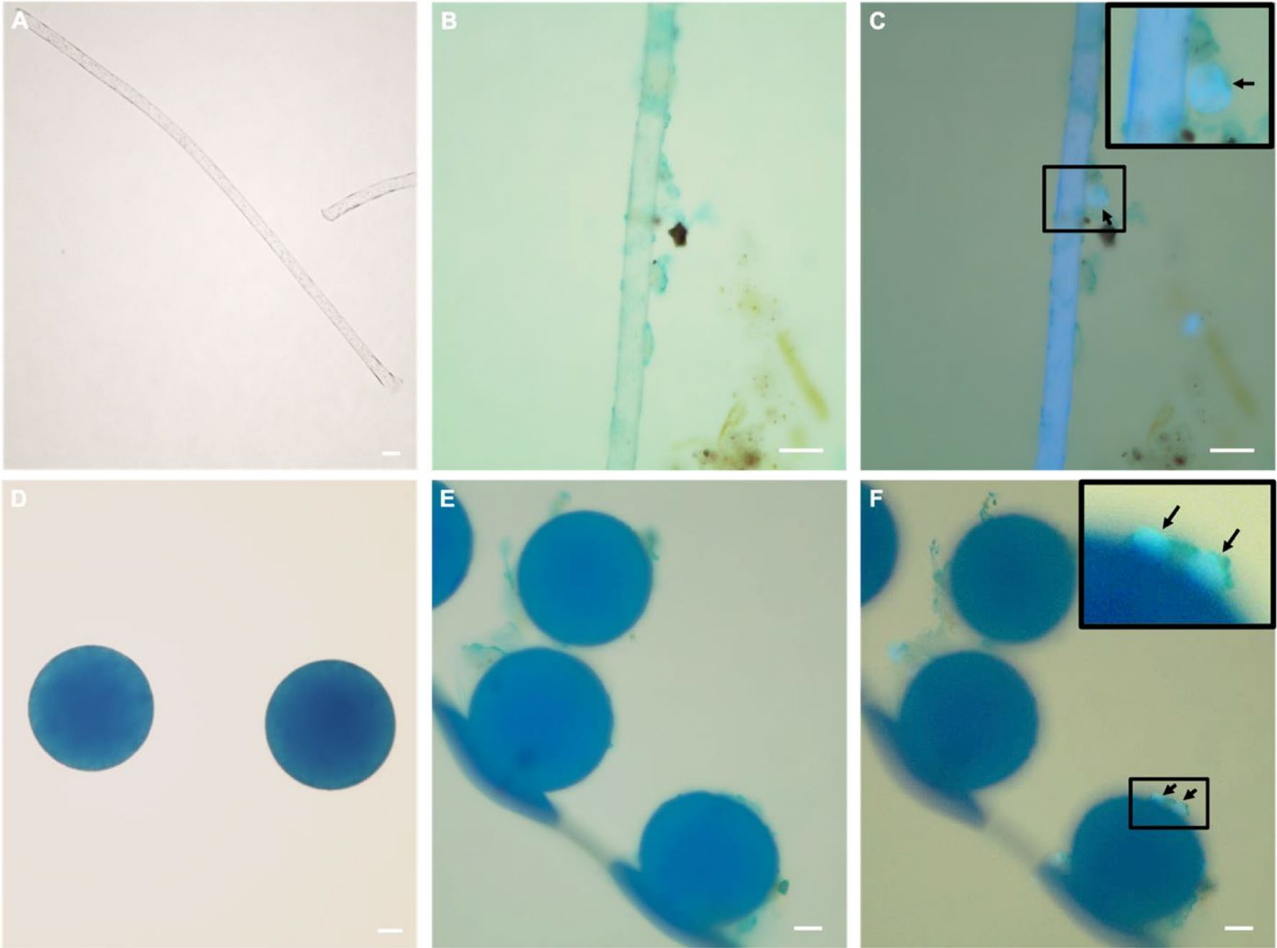
Micrographs depicting the association of *Toxoplasma gondii* with microplastics. Alcian blue, a stain that binds to exopolymer substances prevalent in biofilms, was used to visualize the association between the plastic surface and parasite oocysts. Polyester microfibers (**A**) and 100 μm blue polyethylene microbeads (**D**) that were not pre-conditioned in seawater demonstrate a lack of biofilm on the plastics prior to the experiments (no visible light blue matrix on their surface under brightfield illumination). Microfibers and microbeads following pre-conditioning and incubation with *T. gondii* were imaged under brightfield illumination (**B** and **E**, respectively) and a combination of brightfield illumination and UV epifluorescence that allows visualization of naturally autofluorescent *T. gondii* oocysts (**C** and **F**, respectively). Enlarged inset shows a *T. gondii* oocyst embedded in biofilm (blueish irregular matrix) on the fiber and bead surface. All scale bars are 20 μm.

The second experiment focused on a side-by-side comparison for evaluating the degree to which selected protozoan pathogens associated with different sizes and types of microplastics. The highest numbers of *G. enterica* and *C. parvum* (oo)cysts were observed on the large microfibers (Fig. 2B,C). For *C. parvum,* the small microfibers had the lowest parasite count, but the difference was not statistically significant when compared to both sizes of the microbeads (Fig. 3C). For *T. gondii,* the highest number of oocysts were associated with the 500 μm microbeads, however this total oocyst number was not significantly different from oocysts associated with the small or large microfibers (Fig. 2A). Overall, more protozoan (oo)cysts tended to associate with the large microfibers as compared to the other microplastic types. The microbeads and small microfibers were comparable in their ability to associate with (oo)cysts, with the exception of *G. enterica* where there was a statistical difference between the small microfibers and the microbeads (Fig. 2B).

## Discussion

Findings in this study support our hypothesis that parasites can associate with microplastics in contaminated seawater. Given reports of microplastics in multiple fish and shellfish species, our results are particularly timely as they provide novel data suggesting that microplastics may facilitate pathogen entry into marine food webs^3–5^. Previous research has demonstrated that bivalves are more likely to ingest nanoparticles that are incorporated within aggregates compared to nanoparticles that are free floating^28^. And so, pathogens incorporated within a biofilm community on the surface of microplastics may have a higher chance of being ingested by filter feeding marine invertebrates that may not be able to capture and retain a pathogen freely floating in surrounding seawater. These particles may also affect the transport of pathogens in the marine environment depending on whether they sink or float. Microplastics that float along the sea surface will travel large distances and may facilitate the dispersal of pathogens to locations distant from their original terrestrial source. On the other hand, particles that sink may concentrate these pathogens in the benthos where many filter feeding marine invertebrates reside, further increasing the likelihood of pathogen ingestion by these species.

The waterborne pathogens we chose in this study are particularly relevant for both human and wildlife health. In humans, *Cryptosporidium* and *Giardia* spp. cause gastrointestinal disease that can be deadly in young children and immunocompromised individuals^29^. *T. gondii* causes life-long infections in people due to parasite encystation in muscle and brain tissues. If the individual becomes immunocompromised later in life, the parasite may reactivate causing disseminated toxoplasmosis that can be fatal^30^. If pregnant women experience acute exposure, the parasite can cross the placenta leading to infection of the fetus which can cause developmental disorders, miscarriages, or abortion^31^. These zoonotic pathogens are also pertinent to wildlife health. *Cryptosporidium* and *Giardia* spp. as well as *T. gondii* can infect virtually all mammals. *T. gondii* infections have been reported in numerous marine mammal populations, including in a large proportion of the threatened California sea otter (*Enhydra lutris*) population that serves as a keystone species in California coastal ecosystems^32,33^. Infection in sea otters can cause a fatal disseminated disease as well as reproductive sequelae such as abortions in pregnant sea otter dams^32,33^. *T. gondii* also causes mortalities in critically endangered wildlife including Hector’s dolphins and Hawaiian Monk Seals^34,35^.

In our first set of experiments, we investigated whether 500 μm microbeads and 800–1200 μm microfibers can associate with terrestrial protozoan pathogens and whether this association changed over time. We found an increase in parasite counts associated with microplastics over the 7-day experimental duration, with a corresponding decrease in parasite counts in the surrounding seawater. This trend was not as profound for *C. parvum* and may be attributed to the relatively lower recovery of the parasite during our experimental procedure (Tables S1, S2). While the difference in parasite counts associated with the microplastics compared to counts in the seawater may not seem large, one aspect to consider is that the total mass of microplastics occupied a much smaller volume within each experimental unit (bottle) as compared with the seawater volume. When considering the data on a parasite concentration basis, there were orders of magnitude more parasites per gram of plastic as compared to parasite concentrations per equivalent mass of surrounding seawater in our experimental bottles (Fig. S1). Similarly, under environmental conditions, microplastics in a body of water comprise a relatively small proportion of the aquatic environment as compared with the volume of seawater they are suspended in. And thus, the observation that these parasites can become associated with microplastic surfaces is in itself a significant discovery.

In our second set of experiments, we investigated the effects of microplastic type and size on the ability of plastic particles to associate with parasites. Microfibers in the marine environment can result from disintegration of fishing nets as well as from overland runoff or wastewater effluents delivering washing of textiles into marine waters^36–38^. In this study, microbeads were used to represent primary microplastic spheres found in the environment. In general, higher numbers of protozoan parasites were associated with surfaces of the large microfibers as compared with microbeads. Overall, the smaller particles did appear to associate with fewer parasites, likely due to their smaller surface area where surface adhesion with parasites can occur. Interestingly, the microplastic particles with the largest surface area (500 μm microbeads) did not consistently have the highest numbers of associated parasites (Fig. S2). This observation suggests that there are likely other factors in addition to surface area (e.g. surface roughness, plastic chemical characteristics, biofilm composition, etc.) that mediate the degree to which zoonotic protozoan pathogens can associate with plastics. The observation that microfibers demonstrated a greater ability to associate with parasites in surrounding seawater compared to microbeads is significant because microfibers are often the most common microplastic type found in field investigations^39–41^. Microfibers have also been detected in multiple fish and shellfish species^42–45^. Microfibers may thus play a particularly important role in mediating the transport of protozoan pathogens in marine ecosystems. The enhanced association of pathogens with microfibers may be due to the heterogeneous and rough surface of fibers as compared with the smooth microbeads. Studies conducted on biomedical devices used in human medicine have found that surface roughness of a material is a factor that can induce a greater biofilm formation^46^. However, other factors such as hydrophobicity also play a role in biofilm formation and may outweigh the effects of surface roughness^46^. Polyethylene is more hydrophobic in nature than polyester, which is demonstrated by higher sorption of hydrophobic organic contaminants^47^. In future studies, using recovered plastic debris from marine waters may provide further insight on the ability of microplastic fragments to associate with pathogens in surrounding seawater.

While the present experiments were not designed to specifically test for mechanisms of pathogen association with microplastic surfaces, our microscopy observations suggest that sticky biofilms that form on plastics in seawater are important in mediating protozoan pathogen-plastic association (Fig. 3). The technique we used for visualizing protozoa-plastic association was only possible for *T. gondii* oocysts due to their natural autofluorescence under UV excitation^48^. Alcian blue was used as a staining dye because it allows for visualization of the biofilm matrix by staining exopolymer substances that are present as bacterial secretions within biofilms^49,50^. Biochemical substances (e.g. chemicals, nutrients, and sticky polysaccharides) in surrounding seawater that bind to plastic surfaces have been referred to as the ‘ecocorona’ that coat microplastics after their deposition in environmental waters^51^. It is possible that different microplastics used in this study (polyester and polyethylene) form different amounts and/or compositions of substances within the ecocorona that can subsequently influence the degree to which protozoan pathogens are associated with sticky biofilms on plastics. The ecocorona on plastics is critically important to questions on disease transmission because this complex biochemical layer confers surface properties that resemble natural particles recognized by lower trophic species as food, thereby facilitating microplastics (and associated pathogens) uptake into marine food webs^52^. The ecocorona, or biofilms, may facilitate both the ingestion of and translocation of microplastics in biota^53,54^. Future imaging approaches such as confocal microscopy should be explored for characterization and quantification of the biofilms on different types of plastics, as well as better visualization of pathogens embedded within the biofilm. Further research is thus needed to provide insight on how plastic type, surface area, shape, and size affect the ecocorona characteristics, biofilm formation and subsequent pathogen association.

Our experiments were designed to test a proof-of-concept hypothesis that terrestrially derived zoonotic parasites can associate with microplastic pollutants in seawater. We conditioned the microplastics in raw seawater for two weeks prior to addition of parasites to induce some biofilm formation that would occur under natural conditions in coastal waters. In reality, these plastic pollutants exist in the environment for much longer, months to even years. Our 2-week conditioning period and 7-day parasite incubation period may thus underestimate parasite-plastic interaction under natural conditions in the environment. Another point of consideration is the number of (oo)cysts used in the experiments. We used 1000 (oo)cysts in each treatment jar, which is likely higher than the concentration of these pathogens in the marine environment. We chose to use this number of (oo)cysts to ensure that we can get quantitative data in both fractions while still allowing for some loss during processing and pathogen detection methods.

## Conclusion

Here, we provide novel data suggesting a previously unforeseen role of microplastics in mediating the ecology of terrestrially derived pathogens in the marine environment. Microplastics may increase the bioavailability of pathogens by increasing the likelihood of ingestion by lower trophic species such as bivalves. Plastic particles that preferentially sink may result in a concentration of pathogens in the benthos, leading to an increased risk of contamination of benthic invertebrates and fish. Conversely, microplastic pollutants that float could facilitate pathogen dispersion over large distances to pristine sites that are located far from terrestrial pollution sources. Future work should focus on live aquaria studies to determine whether bivalves are more likely to ingest zoonotic pathogens when associated with microplastics. This study highlights a novel mechanism by which anthropogenic pollutants may be mediating the transmission of pathogens in the marine environment, with important ramifications for wildlife and human health.

## Methods

All experimental methods were performed in accordance with the guidelines and regulations set forth by the University of California, Davis.

### Study materials

#### Microplastics

Blue polyethylene microbeads (100 μm and 500 μm) were purchased from Cospheric LLC (Santa Barbara, CA). The microbeads used were meant to represent primary microplastic spheres such as pre-production pellets or microbeads found in personal care products, with polyethylene being one of the most common plastic materials used worldwide. To produce microplastic fibers (microfibers), undyed spun polyester fabric (Item #1414005) was purchased from Testfabrics Inc (West Pittston, PA). Threads of the fabric were separated then cut by hand into microfibers using fabric scissors, a dissecting microscope, and millimeter graph paper to approximate sizes. Microfibers were cut into two size ranges: 400–700 μm and 800–1200 μm. Microplastic fibers in the marine environments are often generated from fishing nets and textiles as a result of washing. These particle sizes were chosen because fish and shellfish have been documented to ingest particles of these size and these microplastic types have been documented in seafood intended for human consumption^55,56^. To quantify the number of microfiber particles per gram of microfibers, the particles were weighed, suspended in ultrapure water, then transferred to a petri dish and manually counted using a dissecting microscope. Guidelines from Cospheric LLC were used to determine the number of microbeads per gram. Prior to the experiments, microplastics were suspended in 5 ml of 0.1% filtered Tween 80 (Fisher Bioreagents) to reduce plastic aggregation. Tubes were vortexed for 30 s then centrifuged at 3000×*g* for 5 min. Following centrifugation, the tween solution was aspirated out and the microplastics were placed in clean beakers containing seawater as described below.

#### Seawater

Seawater was collected from a coastal site that serves as an important region for commercial oyster production in the west coast of the United States (exact location kept confidential to protect growers’ anonymity). The seawater that we obtained was collected from an intake pipe that draws raw seawater from approximately 50 m into the bay.

#### Parasites

*Giardia enterica* stocks were purchased from Waterborne™ Inc (New Orleans, LA) while *Cryptosporidium parvum* stocks were purchased from the Sterling Parasitology Laboratory at the University of Arizona (Tucson, AZ). *Toxoplasma gondii* oocysts were generously provided by Dr. David Aranz Solis and were generated from experimentally infected cats under the approval and oversight of the Institutional Animal Care and Use Committee at the University of California, Davis. (Oo)cysts used were heat inactivated prior to experiments to reduce biohazard risk for laboratory personnel as described in Shapiro et al.^57^. All experiments involving parasites were conducted using protocols approved by the UC Davis Institutional Biosafety Committee (IBC) following approved Biological Use Authorization in the Shapiro laboratory (BUA # R2779).

### Experiment 1: association of protozoan pathogens with microplastics over time

**Figure 4:**
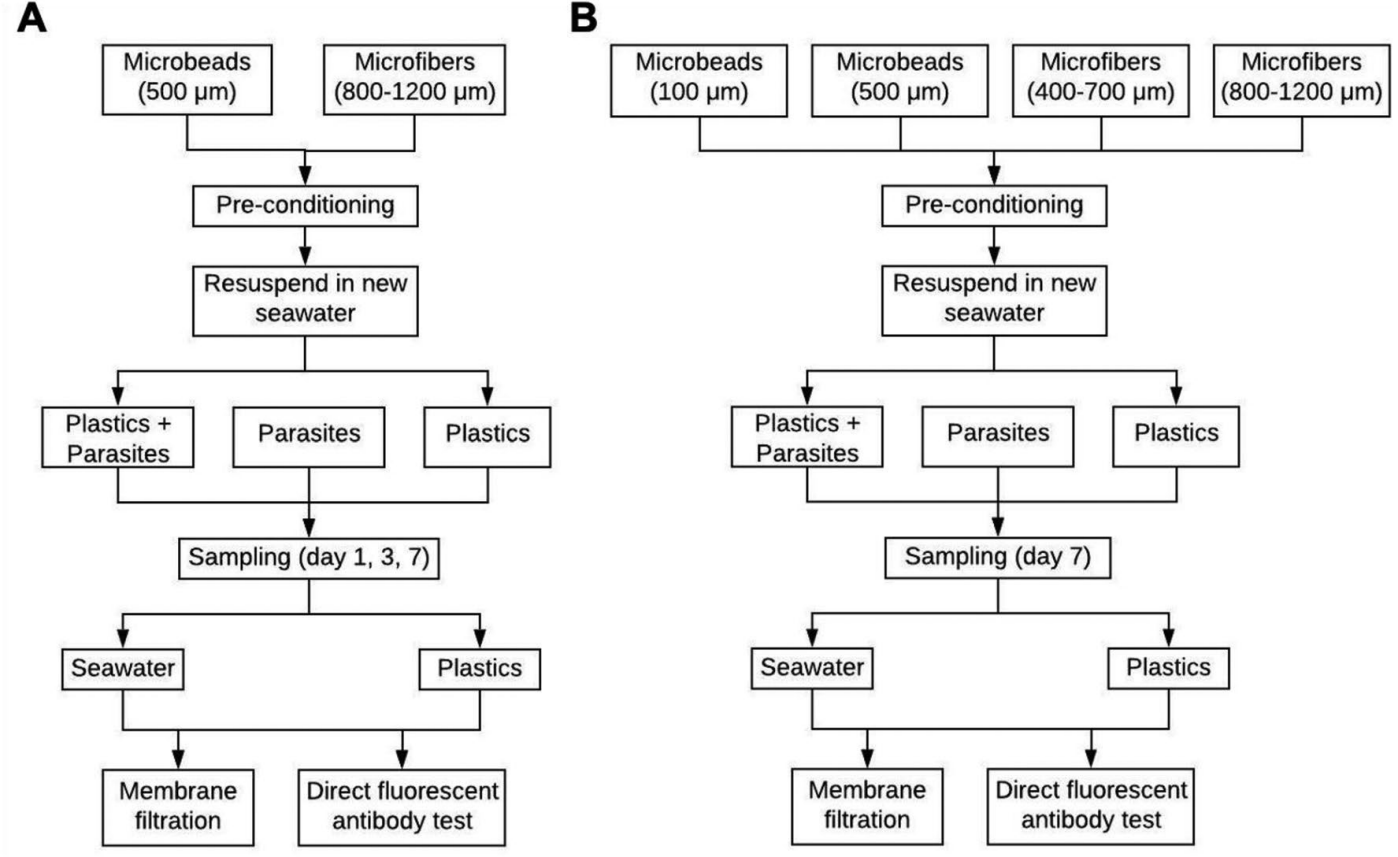
Flow diagram of the experimental design used in Experiment 1 (**A**) and Experiment 2 (**B**). Experiment 1 was designed to determine if microplastics are capable of associating with protozoa in seawater using two microplastic types (microbeads and microfibers), and whether this association changed over time. Experiment 2 was designed to compare the association of protozoan parasites among different microplastic types and sizes.

A flow diagram showing the different steps that were performed to evaluate the association of protozoan pathogens with microbeads and microfibers over time is depicted in Fig. 4A.

#### Microplastic conditioning

To simulate microplastic surface conditioning (i.e. biofilm formation) that occurs when microplastics are present in seawater, we placed microbeads (500 μm) and microfibers (800–1200 μm) in separate 500 ml beakers containing 150 mL of seawater. Beakers were placed on a rocker at room temperature with ambient lighting for 14 days prior to the addition of parasites. Several steps were taken to reduce deposition of microplastics from the surrounding environment in our laboratory. All beakers and treatment jars used for the conditioning period as well as for the microplastic and pathogen incubation were thoroughly cleaned prior to the experiments and then lightly covered to prevent deposition of atmospheric microplastics. Jars were not tightly sealed to avoid anoxic conditions that could affect biofilm formation. When aliquoting seawater, only glass serological pipettes were used. Laboratory personnel wore gloves over the cuffs of their lab coats to prevent microfiber contamination from clothing. Preparation of samples for parasite enumeration was conducted in the fume hood exclusively.

#### Protozoa-microplastic incubation

Following the plastic conditioning period, the beakers were removed from the rocker and placed on the bench for a few minutes to allow the microplastics to settle to the bottom of the beaker. Once the particles settled, a serological pipette was used to gently aspirate the seawater and the settled particles were then resuspended with a new sample of seawater. The beaker was gently swirled so that the microplastics were homogenously suspended before being evenly aliquoted into 100 ml glass bottles with a serological pipette so that every bottle had the same amount (grams) of plastic. The microbeads and microfibers were tested in separate experiments using 30 ml of seawater in each of 27 bottles (15 treatment bottles, 9 plastic-free controls, and 3 negative controls) (Fig. 4A). The treatment and control bottles included the following: microbead treatment bottles (microbeads + parasites), microfiber treatment bottles (microfibers + parasites), no-plastic controls (parasites only), and negative controls (either microfibers or microbeads only). Each treatment was sampled at three different time points. Treatment bottles with microplastics (n = 15, 5 per time point) contained microplastics and a mixture of 1000 (oo)cysts of *T. gondii, G. enterica,* and *C. parvum*.

For the microbead experiment, each bottle contained 0.053 g of microbeads which equates to about 940 particles. For the microfiber experiment, each bottle contained 0.000156 g of microfibers representing about 481 particles. The number of microplastic particles used in our study is higher than previously published reports on microplastic concentrations in the environment—as the aim of our experiments was to design a proof-of-concept experiment to evaluate whether microplastic-protozoa association can be observed under experimental conditions. In our study, we used a concentration of 31 microbeads/mL and 16 microfibers/mL in our first experiments and a concentration of 31 microplastics/mL in the second experiment. Environmental concentrations of microplastics in seawater vary widely depending on the geographical location and sampling approach (e.g., sampling depth)^39,58,59^. In one study reported from North America, microplastic concentrations ranged from 8 to 9180 particles per m^3^ (approximately 8 × 10^–6^ to 9.2 × 10^–3^ particles per mL)^39^. In the present study, we spiked seawater with the lowest possible number of particles that could be used while still being able to visualize them in the treatment jars for the practicality of the described experiments.

Plastic-free controls (n = 9, 3 per time point) contained 1000 (oo)cysts of each parasite in seawater without microplastics. The negative control (n = 3, 1 per time point) contained the same amount of microplastics as the treatment jars with no parasites added. The purpose of the plastic free control was to assess parasite loss and retainment during the post-experimental filtration without the presence of microplastics (described further below), while negative controls served to assess for parasite cross contamination. All bottles were placed on a rocker and sampling was conducted on days 1, 3, and 7.

#### Microplastic separation from seawater

At each sampling point, five treatment jars, three plastic-free controls and one negative control were removed from the rocker for processing. To separate microplastics from surrounding seawater, each bottle was gently swirled to resuspend the microplastics and its contents poured through a cell strainer (100 μm for microbead samples, 40 μm for microfiber samples) into a 50-ml centrifuge tube. A smaller pore size strainer was used for the microfiber experiment to reduce potential loss of the thin fibers through the mesh. One ml of ultrapure water was used for rinsing the strainer to remove parasites that were not associated with the plastics. The jar was then rinsed with 7 ml of ultrapure water to recover any remaining microplastics or parasites and poured through a 2nd cell strainer placed over the same falcon tube. The process was then repeated for a second wash. The filtrate (representing the surrounding seawater fraction) was split for membrane filtration to enumerate *T. gondii* and the direct fluorescent antibody (DFA) test to enumerate *C. parvum* and *G. enterica*.

To recover the parasites from microplastics, cell strainers were inverted over a funnel and washed with 12.5 ml 0.1% Tween into a clean 50-ml centrifuge tube. The tube was vortexed for 30 s and then placed undisturbed to allow plastics to settle to the conical tip. Once the plastics settled, the supernatant was aspirated, and the process repeated using three 5-ml aliquots of Tween washes to maximize recovery of parasites from the microplastic surfaces. The resulting washes represented the fraction of plastic-associated parasites. Similar to the seawater fraction, this plastic wash was split into two samples for membrane filtration and the DFA test.

#### Enumeration of parasites

Enumeration of *C. parvum* and *G. enterica* was completed through Direct Fluorescent Antibody (DFA) staining using the EasyStain™ kit (Biopoint Pty Ltd., SNW, Australia). Seawater or microplastic wash were centrifuged reserving 100 µL pellets that were vortexed and mounted on 3-well SuperStick™ slides (Waterborne™, Inc., LA, USA). Fifty μl of ultrapure water was added to the microcentrifuge tube to resuspend any remaining particles, vortexed and added to the well on the slide. The remainder of the procedure followed manufacturer instructions. To enumerate *T. gondii* oocysts via membrane filtration, samples were filtered through a 25 mm mixed cellulose membrane filter with a 5-µm pore size (Millipore, MA, USA). The membranes were mounted on a glass slide and oocysts were enumerated under UV excitation as described in Shapiro et al.^60^. Membrane filtration and subsequent microscopy combined with staining with Alcian blue, which allowed for visualization of exopolymer substances that constitute the matrix of biofilms, was completed in a small-scale imaging experiment to visualize the protozoa-plastic association. Images were taken using a Zeiss Axioskop epifluorescent microscope equipped with a UV emission filter set.

### Experiment 2: effect of microplastic type and size on protozoa association

In this experiment, our objective was to compare the ability of protozoan (oo)cysts to associate with different microplastic types. Two microplastic types, each with two size ranges, were tested: 100 μm and 500 μm microbeads, 400–700 μm and 800–1200 μm microfibers. Conditioning of microplastics, separation of microplastics from seawater and parasite enumeration were performed using the same methodology as described above. Based on results from Experiment 1 showing that the greatest plastic-parasite association occurred after 7 days of incubation, this second experiment used a single time point (day 7). Combined, there were 6 treatments: 100 μm microbeads + parasites (n = 5), 500 μm microbeads + parasites (n = 5), 400–700 μm microfibers + parasites (n = 5), 800–1200 μm microfibers + parasites (n = 5), no-plastic control (parasites only, n = 3), and a negative control (plastic only, n = 1). Small experimental changes were made for this set of experiments. Microplastics were aliquoted so that the same number of microplastics (940 particles) was used for both microbeads and microfibers, rather than dry weight of each type. The volume of each jar remained the same (30 ml). During the separation of microplastics from the seawater, 40 μm cell strainers were used for all microplastics types. A flow diagram showing the different steps that were performed to compare the association of protozoan pathogens with microbeads and microfibers is depicted in Fig. 4B.

### Data analysis

For each experiment, timepoint and plastic type, parasites recovered from the seawater fraction or the microplastics were analyzed as the total numbers of parasites present in each fraction. In experiment 2 to account for parasite association with organic debris that was retained on the cell strainer in the absence of microplastics, we applied a ‘correction’ step in our data analysis using enumerated parasite numbers from the plastic-free controls: the mean number of parasites recovered from the plastic-free control strainers was subtracted from the microplastic wash samples. This same mean number of parasites was then added to parasite counts in seawater fractions to account for the total number of parasites that were not associated with microplastics. A Mann–Whitney non-parametric test was performed to determine significant differences between total parasite counts in seawater and those associated with microplastics. A Kruskal–Wallis analysis was further applied to test for significant differences in parasite counts associated with microplastics over time (Experiment 1), as well as among different microplastic types (Experiment 2). All statistical analyses were completed using Prism (version 8.4.3, San Diego, CA) with significance threshold determined at *P* < 0.05.

## Supplementary Information

Supplementary Information.

## Data Availability

Additional data are provided in the supplementary data files. Raw data can be further provided upon contacting the authors.
