# Supplementary material for: Association of zoonotic protozoan parasites with microplastics in seawater and implications for human and wildlife health
Source: Sci Rep. 2022 Apr 26;12:6532. doi: 10.1038/s41598-022-10485-5 (PMC9042925; doi:10.1038/s41598-022-10485-5)
Supplement: Supplementary file 1 — Supplementary Information. [file 41598_2022_10485_MOESM1_ESM.docx]

**Association of zoonotic protozoan parasites with microplastics in seawater and implications for human and wildlife health**

Emma Zhang ^1^, Minji Kim ^1^, Lezlie Rueda ^1^, Chelsea Rochman ^2^, Elizabeth VanWormer ^3^, James Moore ^4^, Karen Shapiro ^1,^ *

**Supplemental data**

**Table S1:** Parasite recoveries for the microbead experiments in Experiment 1 (See Fig. 1A). The recovery was calculated as the total numbers of parasites recovered (sum of parasites in seawater and on the plastics) divided by the total number of spiked (oo)cysts (N=1000).

|  | *Toxoplasma gondii* | *Giardia enterica* | *Cryptosporidium parvum* |
| --- | --- | --- | --- |
| Day 1 | 39.6% | 19.2% | 8.4% |
| Day 3 | 38.0% | 19.9% | 9.1% |
| Day 7 | 37.0% | 23.5% | 11.1% |

**Table S2:** Parasite recoveries for the microfiber experiments in Experiment 1 (See Fig. 1B). The recovery was calculated as the total numbers of parasites recovered (sum of parasites in seawater and on the plastics) divided by the total number of spiked (oo)cysts (N=1000).

|  | *Toxoplasma gondii* | *Giardia enterica* | *Cryptosporidium parvum* |
| --- | --- | --- | --- |
| Day 1 | 23.0% | 21.0% | 18.6% |
| Day 3 | 17.4% | 11.3% | 5.8% |
| Day 7 | 30.0% | 18.5% | 6.8% |


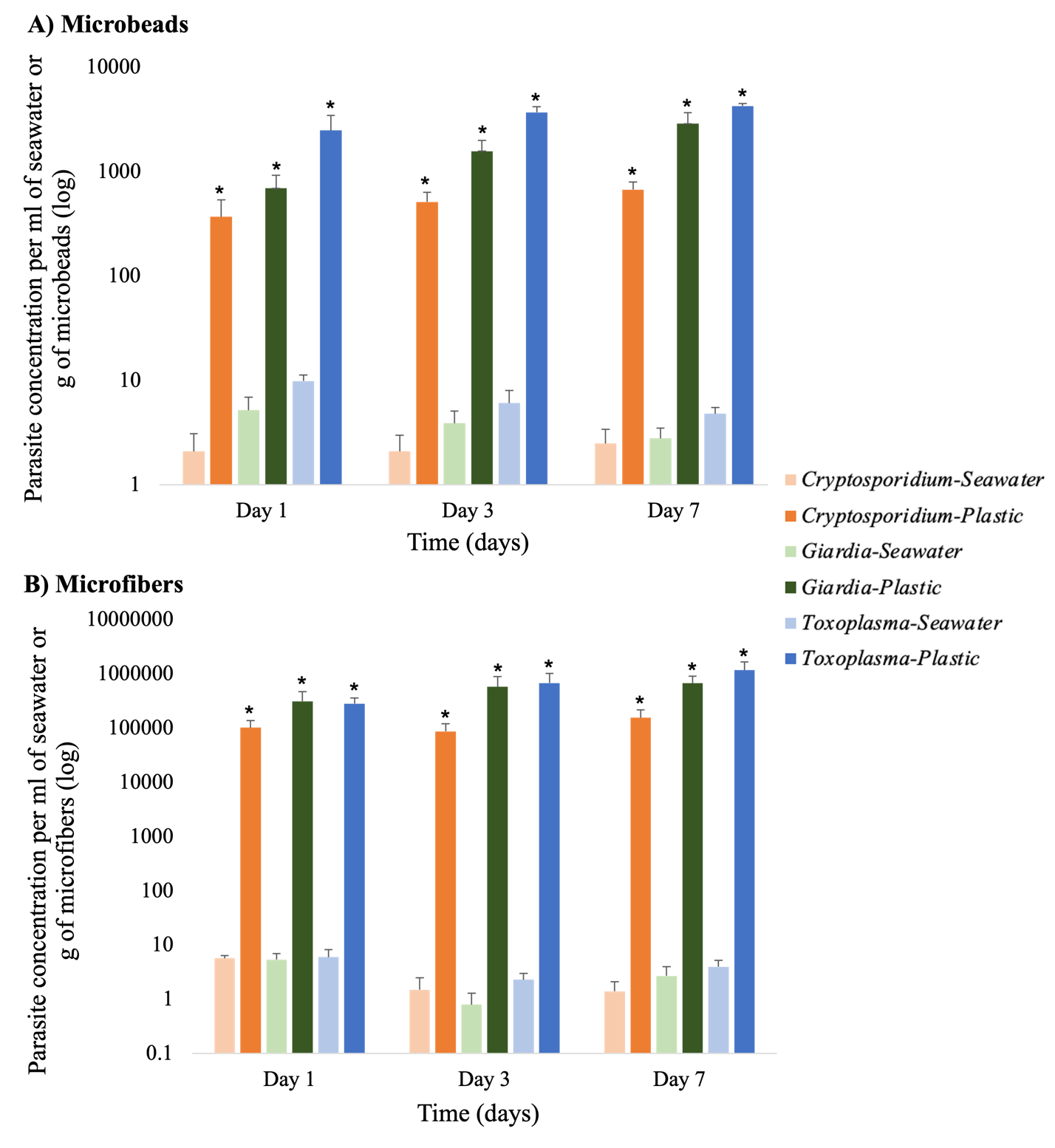


**Figure S1:** Parasite concentration associated with 500 μm polyethylene microbeads (A) or 800-1200 μm polyester microfibers (B) compared to surrounding seawater for Experiment 1 (Fig. 1). Lighter colors represent parasite concentrations in the seawater fraction while darker colors represent plastic associated concentrations. Error bars indicate one standard deviation from the mean. Asterisks indicate significance (*P < 0.05*) when comparing parasite concentrations in seawater vs. associated with microplastics. The concentration of parasites in the seawater fraction was calculated based on the 30 ml volume in each bottle while the concentration of parasites associated with microplastics was calculated based on the grams of microplastics in each bottle.

**
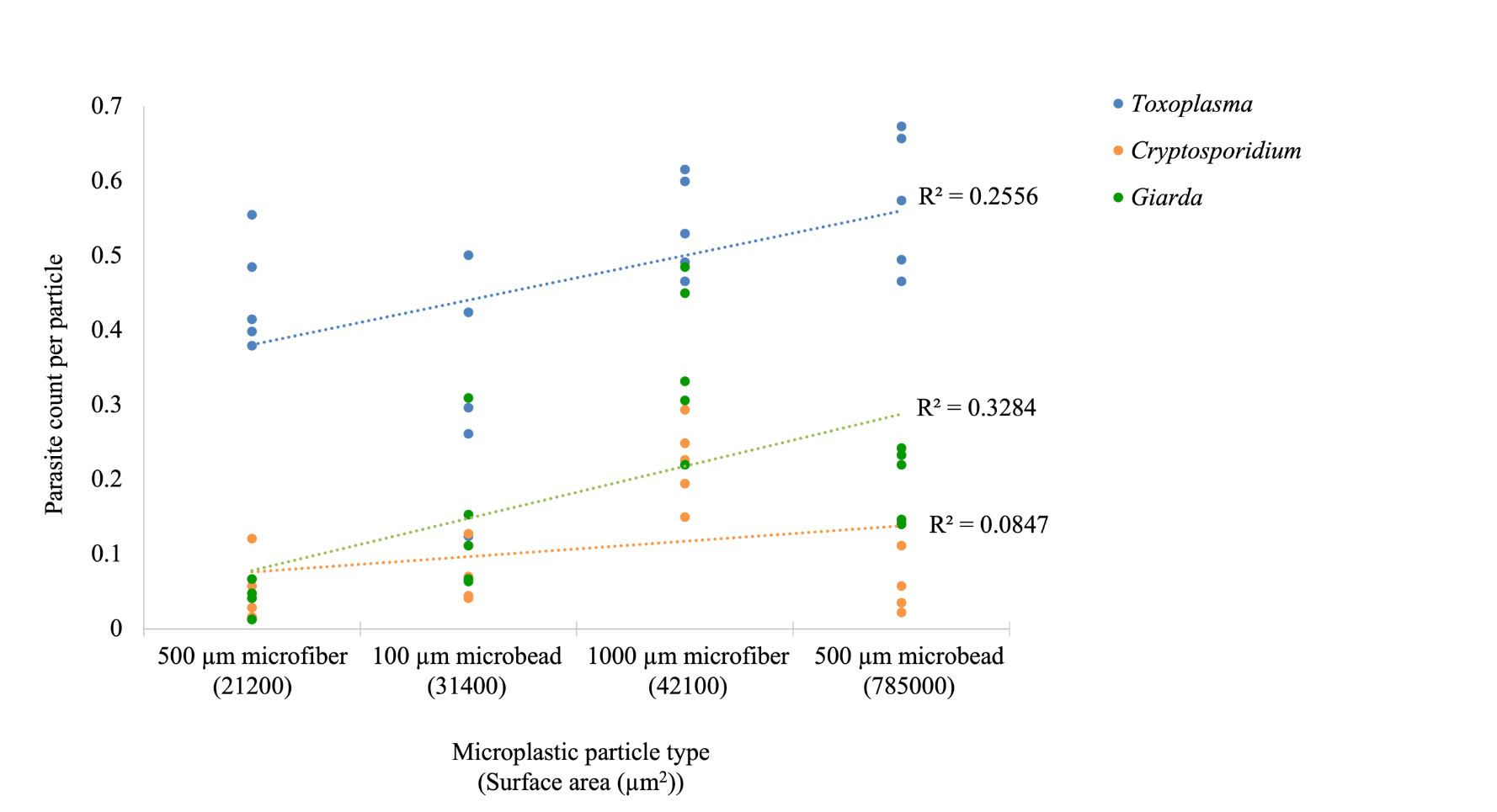
**

**Figure S2:** Experiment 2: Total numbers of protozoan parasites associated with microplastics. In this figure, the microplastics on the X axis are organized in increasing order of total surface area from left to right. Each circle represents a single data point in five experimental replicates for each parasite and microplastic type. Dashed lines represent best fitting linear trend line and associated R^2^ statistic for each protozoan parasite as a function of surface area on the X axis. Intriguingly, while the 500 m beads had substantially larger surface area than other particles, their surface harbored fewer *Giardia* and *Cryptosporidium* (oo)cysts. For the microfibers, the surface area (m^2^) was calculated using an estimated radius of 6.6 μm. For the 400-700 μm microfibers, a length of 500 μm was used for calculation of the surface area while for the 800-1200 μm microfibers a length of 1000 μm was used for calculation. Note that our surface area calculations do not account for pits and crevices in weathered plastic that allow for more areas for growth. Future studies could apply more sophisticated surface area estimations based on scanning electron microscopy to address the relationship between plastic debris surface area and association of harmful pollutants including microorganisms.
